# Moderate evidence for a Lombard effect in a phylogenetically basal primate

**DOI:** 10.7717/peerj.2328

**Published:** 2016-08-16

**Authors:** Christian Schopf, Sabine Schmidt, Elke Zimmermann

**Affiliations:** Institute of Zoology, University of Veterinary Medicine, Foundation, Hannover, Germany

**Keywords:** Lombard effect, Acoustic communication, Vocalization, Voice control, Primate, Mammal, Evolution, Noise, Signal masking, Plasticity

## Abstract

When exposed to enhanced background noise, humans avoid signal masking by increasing the amplitude of the voice, a phenomenon termed the Lombard effect. This auditory feedback-mediated voice control has also been found in monkeys, bats, cetaceans, fish and some frogs and birds. We studied the Lombard effect for the first time in a phylogenetically basal primate, the grey mouse lemur, *Microcebus murinus*. When background noise was increased, mouse lemurs were able to raise the amplitude of the voice, comparable to monkeys, but they did not show this effect consistently across context/individuals. The Lombard effect, even if representing a generic vocal communication system property of mammals, may thus be affected by more complex mechanisms. The present findings emphasize an effect of context, and individual, and the need for further standardized approaches to disentangle the multiple system properties of mammalian vocal communication, important for understanding the evolution of the unique human faculty of speech and language.

## Introduction

A cross-cultural phenomenon of human speech is the Lombard effect (Lombard, 1911), the capability to compensate masking in background noise by adjusting voice amplitude. Recent research suggests that the Lombard effect in humans is sensitive to frequencies important for speech, and not a general response to any competing sound in the environment (Stowe & Golob, 2013). The Lombard effect is thus a complex phenomenon of auditory feedback-mediated voice control (Roy et al., 2011; Eliades & Wang, 2013; Hage & Nieder, 2015).

A feedback-mediated control of voice amplitude has also been described for non-human mammals such as monkeys, whales and dolphins, bats, rodents and cats (Hotchkin & Parks, 2013), and fish (Holt & Johnston, 2014), but not for all studied frogs (Schwartz & Bee, 2013), birds (Schuster et al., 2012) and mammals (Miksis-Olds & Tyack, 2009). In manatees, the Lombard effect depended on the call type and the context, in which the call type was emitted. These conflicting findings in mammals and the fact that non-human mammals have been relatively poorly studied compared to frogs and birds (Hotchkin & Parks, 2013) reflect the need for studies of natural communication situations in other mammalian species.

In primates, the Lombard effect has been studied so far only in monkeys. Macaques of two species (*Macaca nemestrina* and *M. fascicularis*; Sinnott, Stebbins & Moody, 1975) in a restrained and operant conditioning paradigm (monkeys fixed in a primate chair), increased the amplitude of their calls by about 2 dB per 10 dB increase in noise level, if background noise overlapped with the spectral content of their calls. Using a social isolation paradigm, socially-bonded cotton-top tamarins and common marmosets enhanced the amplitude of isolation calls during the playback of band-limited white noise by 2.5–6 dB per 10 dB noise level for cotton-top tamarins (Egnor & Hauser, 2006; Egnor, Wickelgren & Hauser, 2007; Hotchkin, Parks & Weiss, 2015) and 3–7.5 dB for common marmosets (Brumm et al., 2004). No information is available so far for phylogenetically basal primates.

Mouse lemurs are the smallest extant primates and represent unique models for evolutionary and biomedical research due to their basal phylogenetic position within the primates, high cryptic species diversity, and human-comparable brain aging pathology (Martin, 1990; Languille et al., 2012; Zimmermann & Radespiel, 2014). Brainstem-evoked response audiometry revealed that the gray mouse lemur (*Microcebus murinus*) perceives frequencies in the range between 800 Hz and 50 kHz, a frequency band of hearing shifted to the high frequency/ultrasonic range with broader frequency bandwidth than monkeys, apes and humans (Schopf et al., 2014). This broad auditory sensitivity and the highly mobile, bat-like pinnae are important sensory substrates for sound detection, localization and discrimination. Gray mouse lemurs exhibit the broadest distribution of all known 24 mouse lemur species (Hotaling et al., 2016), adapting to various ecological settings in the dry deciduous forests of western Madagascar. They forage solitarily in their dense and three dimensional environments, but also form long-term kin-related social networks (Kessler et al., 2016), relying strongly on acoustic and olfactory cues for prey and predator detection (Scheumann, Rabesandratana & Zimmermann, 2007; Fichtel, 2012; Siemers, 2013) and social communication and decision making (Braune, Schmidt & Zimmermann, 2008; Kessler et al., 2012; Zimmermann, 2016). Gray mouse lemurs are highly vocal and exhibit an elaborate vocal repertoire (Zimmermann, 2016; Zimmermann, in press). The most commonly used call types within their repertoire are the short whistle call and the trill call (Zimmermann, 1995; Zimmermann, 2009; Zimmermann, 2016). Short whistle calls are usually produced in a disturbance context, e.g., when detecting a potential ground predator or an unknown conspecific (Scheumann, Rabesandratana & Zimmermann, 2007; Rahlfs & Fichtel, 2010) and may be classified as a general disturbance, alarm or recruitment call. Trill calls are emitted by mouse lemurs in the breeding season during male courtship approaches, i.e., at a close range to a female, or if she suddenly gets out-of-sight (Zimmermann, 2016). These calls may be classified as a general courtship, mating or advertisement call. Species-specific differences in the acoustic contour of this call type and species-specific recognition support the role of this call type in sexual selection and speciation (Braune, Schmidt & Zimmermann, 2008; Zimmermann, 2016).

**Figure 1:**
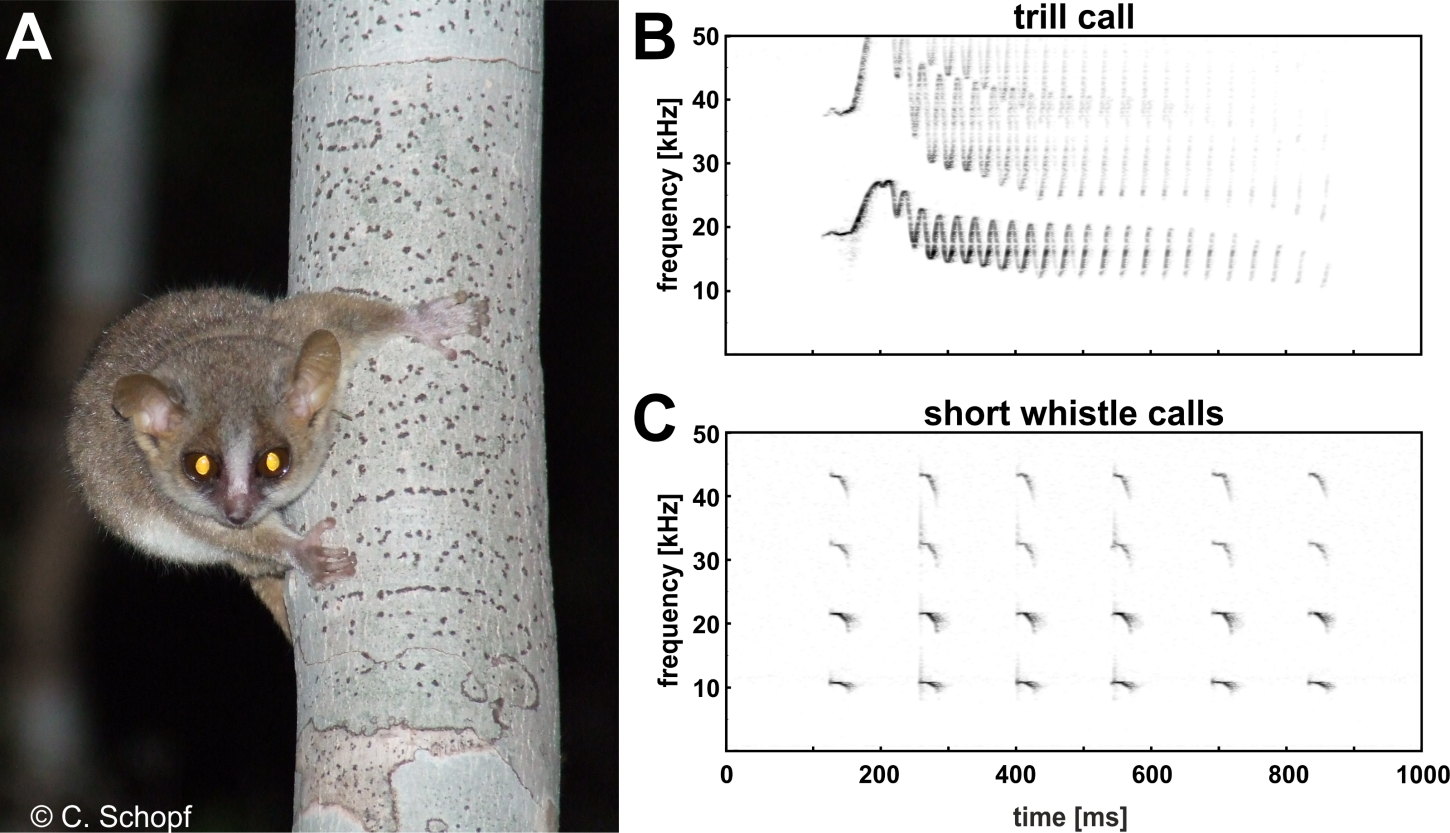
The gray mouse lemur (*Microcebus murinus*, A) and the high frequency/ultrasonic trill call (B) and high frequency short whistle calls (C) displayed as sonagrams.

Ambient noise such as wind, cicadas, crickets and bats are known to overlap the spectral content of communications calls in the natural forest environment of mouse lemurs in Madagascar (C Schopf & E Zimmermann, pers. obs., 2010) and thus may distort signal transmission, even in the high frequency/ultrasonic range (frequency range above 10 kHz). Consequently, we expected that mouse lemurs are able to modify voice amplitude related to high frequency ambient noise covering their communication frequencies, to increase signal-to-noise ratio and thereby the likelihood that others will hear their calls at a distance. To get first insight into auditory feedback-mediated control of voice amplitude in this basal primate, we tested naturally behaving gray mouse lemurs in a typical standardized social encounter situation in the laboratory, and explored (i) whether this nocturnal, and highly vocal, species exhibits the Lombard effect and (ii) whether this effect is present across two spontaneously uttered call types of the vocal repertoire, the high frequency short whistle call, and the high-frequency/ultrasonic trill call (see Fig. 1).

## Material and Methods

### Animals and housing conditions

Twelve gray mouse lemurs were used as subjects. Data from three male and three female subjects, not previously housed together in the same cage, but maintained in the same room, were included in the analysis. All animals reacted by retreating to their nest boxes when being first exposed to enhanced background noise, suggesting noise perception. Normal hearing was experimentally confirmed for two of the subjects in a brainstem-evoked response audiometry study (Schopf et al., 2014).

As described in detail in a previous study (Joly et al., 2014), mouse lemurs of the Hannover breeding colony were kept at the Animal House of the Institute of Zoology, University of Veterinary Medicine Hannover, licensed for the maintenance and breeding of mouse lemurs (Erlaubnis gemäß § 11 Abs. 1 Satz 1 Nr. 1 Tierschutzgesetz in Verbindung mit § 12 Tierschutz-Versuchstierverordnung, Landeshauptstadt Hannover, reference number AZ 42500/1H, 15.01.2014). The mouse lemurs live under a reversed, seasonally fluctuating light cycle (LD 14:10 in long-day period of 8 months, and LD 10:14 in short-day periods of 4 months) and are housed in different rooms where the dark phase, i.e., activity period, started either at 10:00 am, 12:00 am, or 2:00 pm. In all rooms, the temperature and relative humidity are controlled and set to 25 ± 3 °C and 40 ± 10%, respectively. Three times a week, the diet of the mouse lemurs consists of seasonally changing fresh fruits and vegetables, dried fruits, nuts, as well as mealworms or locusts. Milk porridge enriched with vitamins, minerals, and albumin is offered on the other 4 days of the week (see Zimmermann et al. (2016) for further details on life history and maintenance). During the study period, the tested mouse lemurs were maintained either alone or in same-sexed groups in cages of at least 0.75 m^3^ per animal. For this study, mouse lemurs were placed in experimental cages, as described in Experimental Procedure. Wild mouse lemurs are solitary foragers and separation from their sleeping partners and meeting unknown conspecifics temporarily during their activity phase corresponds to their natural behaviour (see also Joly et al., 2014; Lehman, Radespiel & Zimmermann, 2016).

**Figure 2:**
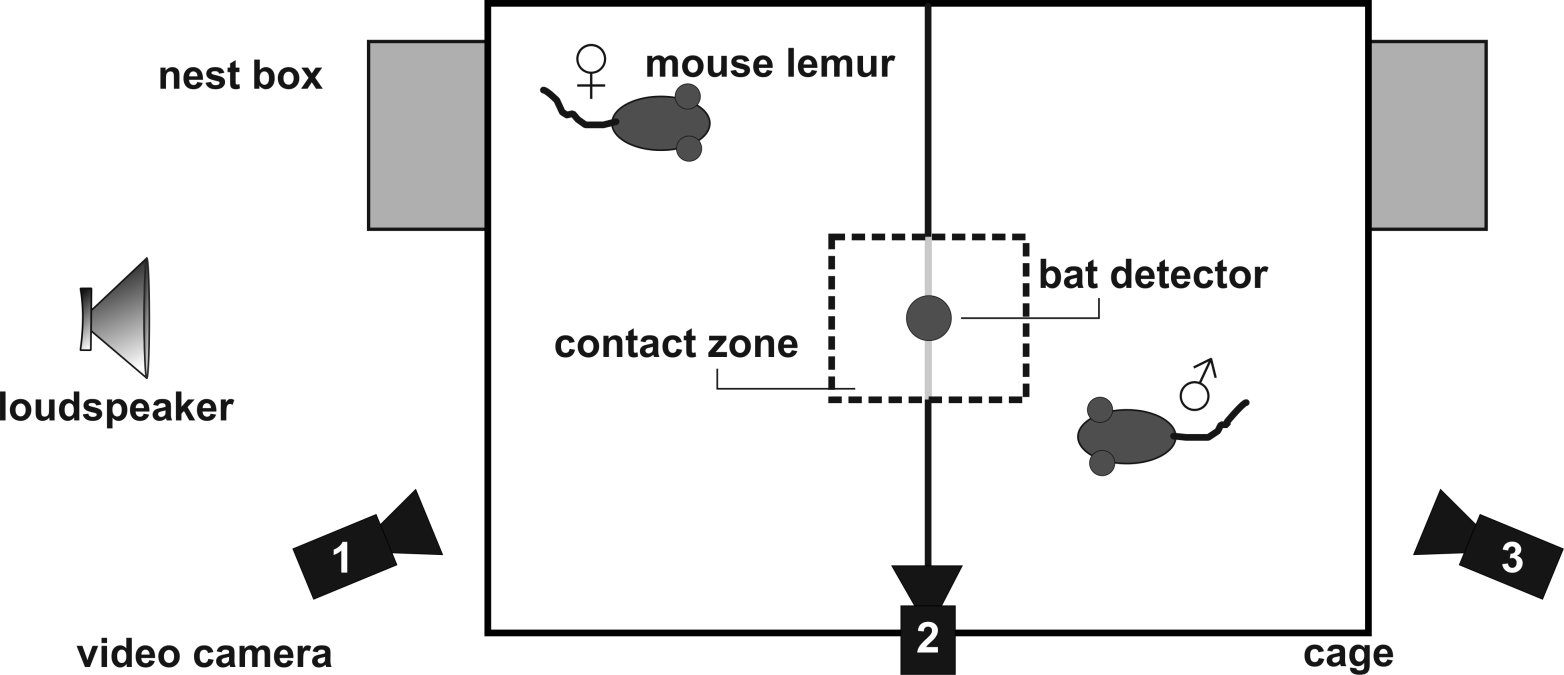
Set-up for the social encounter experiments.

### Experimental procedure

Calling was elicited under standardized conditions by using a social encounter paradigm (Zimmermann, 2009). In the breeding season, an adult male and female were placed adjacently, but separated from each other, in a two-compartment experimental cage (wire-mesh cage, compartment 47 cm × 47 cm × 26 cm, Fig. 2) in a sound-attenuated chamber. This condition generally induces trill calling in male, and short whistle calling in female mouse lemurs. A metal grid, covered with thin white cloth, separated the compartments. A cloth-free window of 12.5 cm × 13.0 cm was left for visual contact (contact zone). Calls emitted during an experiment were recorded by a Pettersson D1000X ultrasound detector with a 16-bit resolution and a sampling rate of 250 kHz. The microphone of the ultrasound detector was positioned 40 cm directly above the center of the test cage to minimize variation in sound level caused by lateral head movements of the subjects (see Brumm et al., 2004). All experiments were videotaped using three digital camcorders (Sony DCR-SR35) in night shot mode.

**Figure 3:**
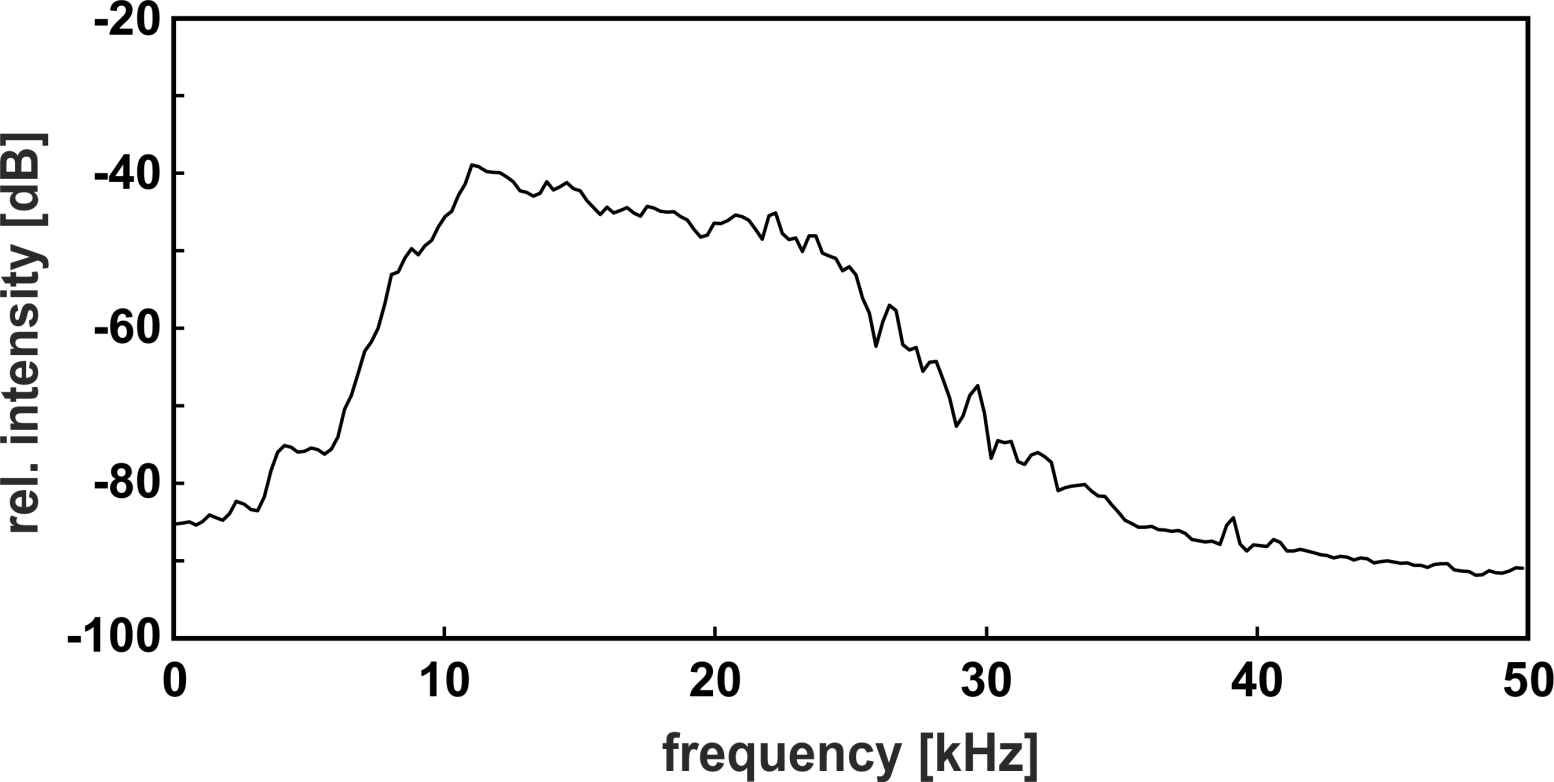
Power spectrum of the band-pass filtered noise. Please note the moderate noise slope, which covered the relevant components of both call types within the auditory range of the gray mouse lemur.

Band-pass filtered white noise with lower and upper cutoff frequencies of 12, and 26 kHz, respectively, was produced by a noise generator (Brüel & Kjær Type 1027) connected to a dual hi/lo filter (Rockland model 852; frequency response see Fig. 3) and played back at levels of 60, 65, or 70 dB SPL via an amplifier (harman/kardon, HK 980) and a high frequency loudspeaker (quadral ribbon tweeter 923108) placed at a distance of 150 cm from the back side of the male’s compartment. The noise production system was powered on, but silent, during the “no-noise” condition. Without additional noise playback, i.e., when the input of the amplifier was grounded, the ambient noise level in the sound-attenuated chamber was below 35 dB SPL in the range of 12–26 kHz. We measured the sound pressure levels of noise in the center of the male’s section of the cage by a free-field ¼ inch microphone (Brüel & Kjær Type 4939 with preamplifier Type 2670) connected to a measuring amplifier (Brüel & Kjær Type 2610; time constant ‘fast’; linear frequency weighting; measuring range 22.4–200,000 Hz). Sound pressure levels in the corners of the cage varied by ±2 dB compared to the center.

Before the first experiment started, each test pair was habituated to the experimental set-up in 20-min habituation sessions. We defined a test pair as habituated when both subjects were active outside the nest box and emitted calls. Test pairs needed three to four habituation sessions to fulfil the criterion. After habituation, each test pair completed four experiments of 30 min total duration, with intervals between experiments of at least one day. To control for differences in calling motivation across experimental days, 5-min no-noise, and noise, intervals alternated in each experiment, starting with a no-noise interval. Each of the three noise levels was presented for one 5-min interval per experiment; their presentation order was randomized across the four experiments.

### Acoustic analyses and statistics

The occurrence of calls was analyzed by displaying them visually as oscillogram and sonagram using BatSound Pro 3.0 (Pettersson Elektronik AB, Uppsala, Sweden). Call types were identified according to Zimmermann (2009). Caller identification was achieved by synchronizing the audio and video recordings and checking in the video which animal had emitted the call. Movements of the mouth, flanks, or a combination of both were used to detect call emission (for method see Zimmermann, 1996).

Trill calls and short whistle calls assigned to the respective caller were analyzed if they were not overlapping with calls of the pair partner, or movement noise, and if the caller was oriented towards the interaction partner, i.e., facing the separation grid, in quadruped position with the head held horizontally. To account for position-related call amplitude differences within the experimental compartment, we played back a trill call, and a short whistle call series, from 15 equally spaced positions of the test cage and corrected for the obtained amplitude differences relative to the position directly under the microphone. Avisoft-SASLab Pro software was used to measure the level of recorded calls adopting the procedure used by Brumm et al. (2004). Avisoft was calibrated using 2-s noise segments of the three noise conditions, respectively, recorded directly at the position of the ultrasound detector microphone. Afterwards, the maximum root-mean-squared sound pressure value of the call with an averaging time of 1 ms was measured. Finally, we determined the sound pressure level of each call using the logarithmic computation procedures described in Weißing (1984).

Six subjects produced calls in the condition without additional background noise (no-noise condition) and in either 60, 65, or 70 dB SPL background noise (noise condition, see Table 1). Two of the subjects were paired, the other four subjects had interaction partners whose vocalizations did not meet the above described criteria for analysis. Noise-dependent differences in call amplitude of the respective call type between the noise and no-noise condition were explored for each subject using two-sided Mann–Whitney *U*-tests with *α* set at 0.05. Tests were performed using Statistica 6.1 (StatSoft).

**Table 1:** Medians and quartiles for amplitude measurements of mouse lemurs in the no-noise and noise condition for trill calls (M, male) and short whistle calls (F, female).

Subject	No-noise				Noise				MWU	
	*n*	Median (dB)	Lower quartile	Upper quartile	*n* (masking noise in dB)	Median (dB)	Lower quartile	Upper quartile	U	P
M1	9	65	61	89	3 (60)	97	64	98	5	n.s.
M2	9	63	62	69	10 (60)	62	60	70	39	n.s.
M3	6	62	58	65	8 (60)	64	61	69	17	n.s.
F1	8	80	69	87	12 (65)	94	92	96	5	^**^
F2	18	73	71	77	35 (60)	85	83	87	12	^***^
F3	7	87	86	92	6 (70)	84	83	86	10	n.s.

**Notes.**

** *P* < 0.00343.

*** *p* < 0.0000001.

### Ethics

Experiments are non-invasive and belong to basic research. Experiments were performed in accordance with the NRC Guide for the Care and Use of Laboratory Animals, the European Directive 2010/63/EU on the protection of animals used for scientific purposes, and the German Animal Welfare Act as well as the national guidelines of the German Society of Primatology (GfP) for research on non-human primates. The non-invasive procedure was approved by the animal welfare officer of the University of Veterinary Medicine Hannover, Foundation as well as by the State of Lower Saxony Office for Consumer Protection and Food Safety (LAVES; approval date: October 20, 2010; number: 33.9-42502-05-10A080), which is the responsible agency of the State Lower Saxony for approval of animal studies according to the German Animal Welfare Act (TierSchG). All information mentioned is in accordance with the recommendations of the Weatherall report, “The use of non-human primates in research”.

We provided environmental enrichment to the mouse lemurs: within the housing cages, the animals of our colony are provided with branches and hollow cylinders that allow the animals to climb and hide within their home cages. To model the situation in nature, where mouse lemurs sleep, rest and rear their offspring in tree holes (e.g., Radespiel, Ehresmann & Zimmermann, 2003), each cage is equipped with several sleeping boxes.

## Results

**Figure 4:**
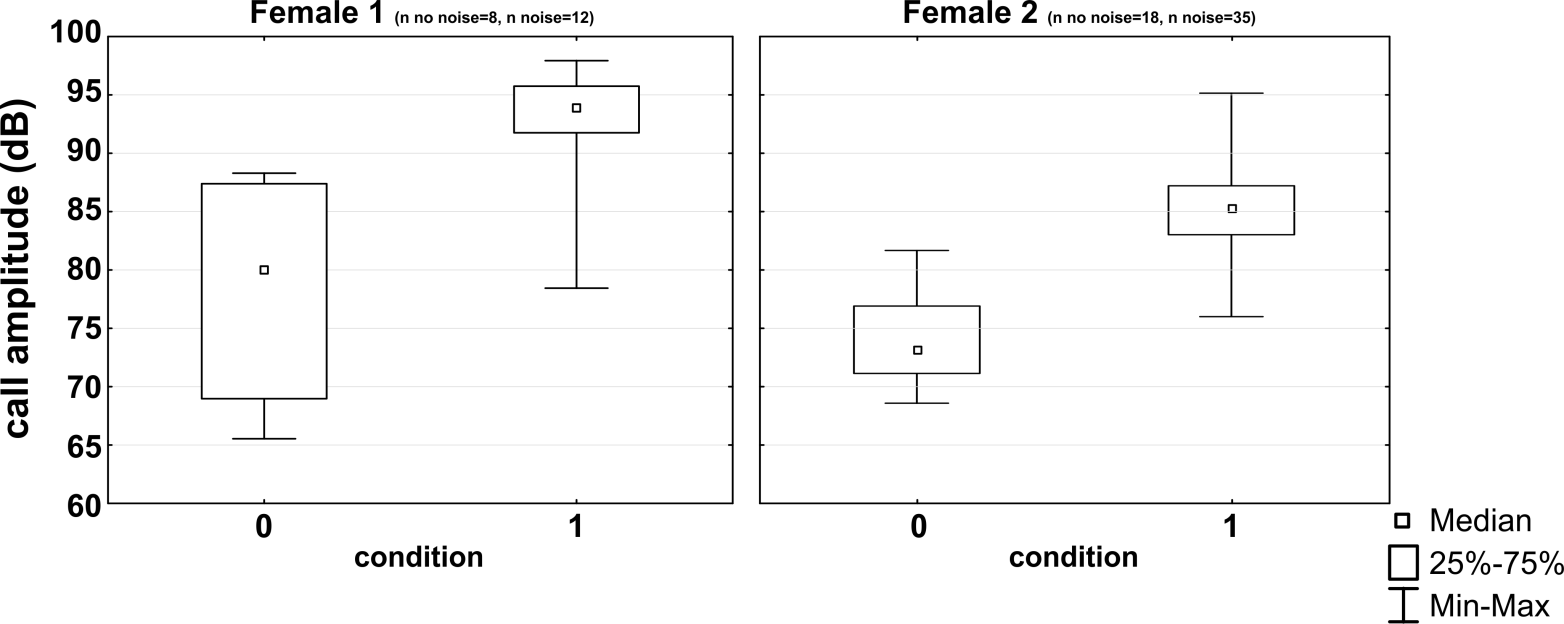
Noise-dependent changes in call amplitude. Medians, interquartile ranges and maximum and minimum values are given for the no noise (0) and the noise (1) condition.

All subjects experienced all noise levels, but they did not emit analyzable vocalizations in every condition. Three subjects produced short whistle calls (see Fig. 1) and three different subjects trill calls (see Fig. 1) in both, the noise and no-noise condition. Short whistle calls varied between 73 dB SPL and 87 dB SPL across subjects in the no-noise and between 84 dB SPL and 94 dB SPL in the noise condition. In noise, two subjects showed a significant increase in voice intensity and increased call amplitude by either 12 dB or 15 dB (see Fig. 4). Such an effect could not be revealed for the third subject (MWU, n.s.; see Table 1). Trill calls varied between 62 dB SPL and 65 dB SPL across subjects in the no noise and between 62 dB SPL and 97 dB SPL in the noise condition (Table 1). There was no significant noise-dependent difference in sound amplitude of subjects (MWU, n.s.; Table 1).

## Discussion

We provide the first evidence for a moderate Lombard effect in a phylogenetically basal, nocturnal primate. Interestingly, this auditory feedback-mediated control of voice amplitude was not consistently present across the studied call types and subjects.

As we expected, the gray mouse lemur is able to adjust the voice amplitude of the short whistle call, i.e., the high frequency general disturbance call, when exposed to background noise, covering the call’s spectral content, in line with comparable findings in monkeys, e.g., for different call types given in social isolation by cotton-top tamarins (Egnor & Hauser, 2006; Hotchkin, Parks & Weiss, 2015) and marmosets (Brumm et al., 2004). This auditory feedback-mediated vocal flexibility of a subject may be beneficial since it may enhance its fitness by optimizing sound transmission in a noisy environment, e.g., by advertising the presence of an alerting stimulus to conspecifics. Interestingly, however, only two of three subjects, producing calls in the standardized social encounter paradigm, significantly increased voice amplitude. The third subject, exhibiting a “loud” voice even in the no-noise condition, did not show a noise-dependent amplitude change, suggesting that personality may affect voice modification. In contrast to our hypothesis, mouse lemurs did not show auditory feedback mediated vocal amplitude adjustment in trill calls, i.e., courtship calls, which males usually produce in the breeding season during courtship approaches to females, i.e., at a close range. Our study revealed that neither of the tested subjects did modify voice amplitude significantly when exposed to enhanced background noise. One explanation is that the amplitude of the noise, covering the spectral content of the trill call, is too low for yielding auditory feedback-mediated vocal adjustment. Indeed, background noise amplitude is at maximum in the 12–14 kHz range of the short whistle call (Zimmermann, 2009), and dropped slightly in the broad 10–26 kHz range of the trill call (Zimmermann, 2009). An alternative explanation is that the three subjects may have been at their upper energy limit for the production of this call type, which displays the longest duration within the species vocal repertoire (Zimmermann, 2009) and is involved in sexual selection and speciation (Zimmermann, 2016). It is known that anatomical constraints may limit vocal output and contribute to honesty in animal communication calls (Reby & McComb, 2003). Consequently, subjects should emit vocalizations of similar amplitudes both in the noise- and no-noise condition, or remain mute if the background noise gets too high. This was exactly what we found for the courtship calls in our subjects, which did not emit any call if background noise was higher than 60 dB. This absence of the Lombard effect in a courtship situation furthermore coincides with findings in some frogs (Schwartz & Bee, 2013) and birds (male tinamou, Schuster et al., 2012) and supports the notion that vocal output of subjects in a courtship situation is energetically constrained.

Our data showed moderate evidence for the Lombard effect in a basal primate. In contrast to monkeys, an auditory feedback-mediated voice amplitude adjustment was not found consistently across different call types, but seems to be dependent on context, similar to findings in manatees (Miksis-Olds & Tyack, 2009). From humans it is also known that a noise-dependent regulation of voice amplitude shows individual variation and is linked to factors such as speaker, context, language, linguistic content, or noise type (Hotchkin & Parks, 2013). Recent research on bats and nonhuman primates further provided evidence for auditory mediated flexibility in communication calls (Esser, 1994; Ouattara, Lemasson & Zuberbühler, 2009; Knörnschild, 2014; Watson et al., 2015; Luo & Wiegrebe, 2016). Further standardized approaches to auditory mediated control of voice considering biologically relevant contexts (Hotchkin & Parks, 2013; Choi, Takahashi & Ghazanfar, 2015) and a broader range of taxa are needed to disentangle the multiple system properties in mammalian vocal communication, crucial for understanding the evolution of the unique human faculty of speech and language.

##  Supplemental Information

10.7717/peerj.2328/supp-1 Data S1 Raw data of subjects with their corresponding call types and noise levels

## Additional Information and Declarations

### Data Availability

The following information was supplied regarding data availability: The raw data has been supplied as Data S1.
